# Neurodevelopmental Outcomes after Pediatric Cardiac ECMO Support

**DOI:** 10.3389/fped.2013.00047

**Published:** 2013-12-19

**Authors:** Constantinos Chrysostomou, Timothy Maul, Patrick M. Callahan, Khoa Nguyen, Steven Lichtenstein, Emma G. Coate, Victor O. Morell, Peter Wearden

**Affiliations:** ^1^Critical Care Medicine, Cardiac Intensive Care, Children’s Hospital of Pittsburgh of UPMC, Pittsburgh, PA, USA; ^2^Cardiothoracic Surgery, Children’s Hospital of Pittsburgh of UPMC, Pittsburgh, PA, USA; ^3^Department of Anesthesiology, Children’s Hospital of Pittsburgh of UPMC, Pittsburgh, PA, USA; ^4^Dietrich School of Arts and Sciences, University of Pittsburgh, Pittsburgh, PA, USA

**Keywords:** ECMO, neurodevelopmental outcomes, children, PCPC, E-CPR, cardiac arrest, intracranial hemorrhage, cerebral infarction

## Abstract

**Purpose:** To characterize the neurodevelopmental outcomes and identify factors associated with poor outcomes in pediatric patients undergoing cardiac extracorporeal membrane oxygenation (ECMO).

**Methods:** Five year retrospective review, including demographics, cardiac lesion, and surgical complexity, reason for ECMO, ECMO complications, and neurodevelopmental status at discharge and latest follow-up. Neurodevelopmental status was determined through the Pediatric Overall Performance Category and Pediatric Cerebral Performance Category Scales.

**Results:** Overall ECMO survival was 73% at hospital discharge and 66% a t the latest follow-up. Most patients underwent cardiopulmonary resuscitation (CPR) (43%), and the majority (53%) had a significant disease complexity (Aristotle = 4). Complications occurred in 42% of the ECMO runs, of which 12% were intracranial injuries. At hospital discharge, 75% of patients had normal to mild disability, improving to 81% at 2 years follow-up. At hospital discharge, moderate to severe disability was associated with CPR, plasma exchange or intracranial insults. After discharge, 23% showed improvement in neurologic status and 4% showed deterioration. Cerebral infarction was the only parameter associated with deterioration at the later follow-up stage.

**Conclusion:** Extracorporeal membrane oxygenation was successfully used in children with cardiac disease with 73 and 66% short and long-term survival respectively. Majority of the survivors had normal to mild neurodevelopmental disability and a significant portion showed neurologic improvement by the latest follow-up. Nevertheless, despite the grossly favorable outcomes standardized comprehensive neuropsychological testing is of paramount importance in all these patients.

## Introduction

Extracorporeal membrane oxygenation (ECMO) has been the preferred method of mechanical circulatory support for patients with acute cardiac and/or respiratory failure since the 1970s. Among other indications it is frequently used in patients where cardiopulmonary resuscitation (CPR) is refractory to conventional resuscitation measures (E-CPR); in patients with low cardiac output syndrome due to postoperative myocardial dysfunction, primary myocardial disease, or arrhythmias where further escalation of therapy outweighs the potential risks of ECMO (LCOS-ECMO); and in patients who fail to separate from cardiopulmonary bypass (OR-ECMO). In contrast to our recent publication showing institutional survival of 73%, the expected average survival rates hover between 40 and 50% (1, 2). Complications among the survivors, and in particular neurologic complications, are not infrequent and maybe related to preceding low cardiac output events, cardiac arrest, or due to intracranial hemorrhage. Because the ECMO circuit is a potent activator of the coagulation system, systemic anticoagulation is required. Therefore maintaining a delicate balance between over- and under-anticoagulation is of paramount importance to prevent hemorrhagic and thromboembolic complications, respectively (3–9). According to recent data from the Extracorporeal Life Support Organization (ELSO) registry, approximately 20% of all ECMO patients have some form of neurological complication, and neuroimaging studies performed on ECMO patients have demonstrated a frequency of abnormal results in 30–50% of cases (2, 10). Similar results have been obtained for cardiac ECMO patients, with up to 60% having moderate to severe neurological impairment (11). However, the true nature of neurological injury from ECMO patients is clouded by the possible pre-ECMO hypoxia and by the underlying anatomic, neurologic, and neurodevelopmental abnormalities that may occur to as high as 30–50% of patients with congenital heart defects (CHD) (12–15).

The objective of this study was to assess the short and intermediate term neurodevelopmental outcomes of pediatric cardiac ECMO survivors and to identify factors associated with worse outcomes.

## Materials and Methods

This study was granted exempt status from the University of Pittsburgh Institutional Review Board and was a retrospective investigation of ECMO patients in the cardiac intensive care unit (CICU) at Children’s Hospital of Pittsburgh between January 2006 and December 2010. A team of two cardiac surgeons, three cardiac intensivists, and four cardiac anesthesiologists were involved through the study’s 5-year period. Of the 95 patients undergoing ECMO during this time, 69 (73%) survived to hospital discharge and thus were the subject of this study. All patients were supported with veno-arterial ECMO. As part of the institutional ECMO protocol, all patients received an initial EEG when placed on ECMO and received daily head ultrasounds for at least 5 days until decannulation. A detailed description of our ECMO and E-CPR management protocol, including our protocol to assess readiness for decannulation, was previously published (1).

### Neurologic status assessment

Neurologic assessment was performed using the Pediatric Overall Performance Category (POPC) scale and Pediatric Cerebral Performance Category (PCPC), which assess cognitive impairment and functional (general adaptive or physical) morbidity after critical illness or injury, respectively (16, 17). Each is a six-point graded scale of increasing disability from normal function (score = 1) to death (score = 6) (Tables 1 and 2). As seen in Table 2, the PCPC scores are incorporated in the POPC scale. Differences between baseline and discharge POPC and PCPC scores have been associated with several measures of morbidity including ICU length of stay, total hospital charges, and discharge care needs (16).

**Table 1:** **Pediatric cerebral performance category scale**.

Score	Category	Description
1	Normal	Normal; at age-appropriate level; school-age child attending regular school classroom
2	Mild disability	Conscious, alert, and able to interact at age-appropriate level; school-age child attending regular school classroom, but grade perhaps not appropriate for age; possibility of mild neurologic deficit
3	Moderate disability	Conscious; sufficient cerebral function for age-appropriate independent activities of daily life; school-age child attending special education classroom and/or learning deficit present
4	Severe disability	Conscious; dependent on others for daily support because of impaired brain function
5	Coma or vegetative state	Any degree of coma without the presence of all brain death criteria; unaware, even if awake in appearance, without interaction with environment; cerebral unresponsiveness and no evidence of cortex function (not aroused by verbal stimuli); possibility of some reflexive response, spontaneous eye-opening, and sleep-wake cycles
6	Brain death	Apnea, areflexia, and/or electroencephalographic silence

**Table 2:** **Pediatric overall performance category scale**.

Score	Category	Description
1	Good overall performance	PCPC = 1; healthy, alert, and capable of normal activities of daily life
2	Mild overall disability	PCPC = 2; possibility of minor physical problem that is still compatible with normal life; conscious and able to function independently
3	Moderate overall disability	PCPC = 3; possibility of moderate disability from non-cerebral systems dysfunction alone or with cerebral system dysfunction; conscious and performs independent activities of daily life but is disabled for competitive performance in school
4	Severe overall disability	PCPC = 4; possibility of severe disability from non-cerebral systems dysfunction alone or with cerebral system dysfunction; conscious but dependent on others for activities of daily living support
5	Coma or vegetative state	PCPC = 5
6	Brain death	PCPC = 6

The neurologic assessments were obtained at two separate time points. The first assessment was recorded at hospital discharge and the second at the time of the latest outpatient follow-up and when adequate neurodevelopmental information was available in medical records.

### Data collection and categorization

Data collected included demographic information, chromosomal and major non-cardiac structural abnormalities, cardiac diagnosis, and surgery, Aristotle complexity score, need for re-operation, CPR and ECMO parameters, complications during ECMO, and hospital length of stay.

Patients were divided into three major categories, including E-CPR, OR-ECMO, and LCOS-ECMO. Because of the diversity of cardiac lesions and physiology, patients were grouped into: (a) single ventricle lesions (patients with 1 functional ventricle); (b) two-ventricle lesions; (c) primary myocardial disease (including patients with heart transplantation); and (d) primary pulmonary hypertension.

Complications during ECMO were categorized as follows: (a) brain injury (clinical or electroencephalographic seizures, significant central nervous system hemorrhage including epidural or subdural hematoma, subarachnoid hemorrhage and >Grade I intraventricular hemorrhage, and infarction or diffuse ischemic changes based on ultrasound or CT scan); (b) renal injury (serum creatinine ≥ 1.5 mg/dL or dialysis use via hemodialysis, continuous venovenous hemodialysis or hemofiltration); (c) bloodstream bacterial infection; (d) respiratory complications (ventilator associated pneumonia, acute respiratory distress syndrome, pulmonary hemorrhage, or pneumothorax); (e) cardiac complications (new arrhythmias that required treatment, CPR and inotropic support); (f) gastrointestinal complications [gastrointestinal hemorrhage or hyperbilirubinemia (defined as serum conjugated bilirubin ≥ 2 mg/dL or total bilirubin ≥ 15 mg/dL)]; (g) bleeding (cannulation or surgical site bleeding, hemothorax and hemopericardium requiring intervention); (h) mechanical circuit complications (air emboli, thrombus formation in the circuit, breaks, or leaks developing in any part requiring circuit change and oxygenator failure).

### Statistical analysis

All variables were assessed for normal distribution using the Kolmogorov–Smirnov test. Normally distributed data are presented as mean ± standard deviation. Data that were not normally distributed are presented as median with interquartile range (IQR). Categorical variables are presented as the total number followed by the percentage of the population in parenthesis. Categorical data were compared with the Fisher’s exact test or χ^2^ test where appropriate and continuous data with the Wilcoxon test for 2-group comparison or Kruskal–Wallis test for 3-group comparison. Due to the small number of patients in each individual POPC scale, for statistical purposes and meaningful comparison, patients with a POPC score 1 and 2 were considered one category (Normal – mild disability group), and patients with POPC score between 3 and 6 was a different category (moderate to severe disability group). All statistical analyses were performed with JMP version 9.0 (SAS Institute Inc., Cary, NC, USA).

## Results

Of the 95 patients placed on ECMO between January 2006 and December 2010, 69 (73%) survived to hospital discharge and were the cohort considered in this study. The median age and weight of these patients was 47 days (IQR 8–500 days) and 4 kg (IQR 3–9 kg) respectively. Complete demographics are summarized in Table 3. Most patients in this cohort had undergone CPR and based on the Aristotle level, the majority (53%) of the patients had a significant disease complexity.

**Table 3:** **Demographics**.

	*N* = 69
Age, days	47 (8, 500)
Weight, kg	4 (3, 9)
Corrected gestational age	40 (38, 42)
Sex, M (%)	40 (58)
Cardiac diagnosis
Single ventricle, *n* (%)	13 (19)
Two ventricle, *n* (%)	38 (55)
Primary myocardial disease, *n* (%)	15 (22)
Primary pulmonary HTN, *n* (%)	3 (4)
E-CPR, *n* (%)	30 (43)
OR-ECMO, *n* (%)	24 (35)
LCOS-ECMO, *n* (%)	15 (22)
Cardiac surgery before ECMO, *n* (%)	44 (64)
Aristotle level, *n* (%)
1	3 (7)
2	8 (18)
3	10 (22)
4	23 (53)
Chromosomal/structural abnormalities, *n* (%)	7 (10)

Pre-ECMO and during ECMO characteristics are presented in Table 4. The CPR duration was 40 min (IQR 25–50 min). Though 22% of patients had a lactate > 10 before ECMO only 4% had a lactate > 10 after the initiation of ECMO. At least one complication was documented in 42% of the patients (Table 5). Overall incidence of brain injury was 12%, which included seizures, intracranial hemorrhage, and infarction.

**Table 4:** **Pre and during – ECMO parameters**.

	*N* = 69
CPR duration, min	40 (25, 50)
Categorized arterial blood gas pH
pH	7.39 (7.27, 7.52)
pH < 7.21, *n* (%)	11 (16)
Categorized lactate
Lactate	4 (2, 10)
Lactate > 5, *n* (%)	27 (39)
Lactate > 10, *n* (%)	15 (22)
Epinephrine, μg/kg/min	0.1 (0.03, 0.15)
ECMO flow at 4 h, mL/kg/min	131 (97, 154)
ECMO flow at 24 h, mL/kg/min	115 (82, 143)
Categorized ABG – pH
pH	7.5 (7.33, 7.55)
pH < 7.21, *n* (%)	2 (3)
Categorized lactate
Lactate	3 (2, 5)
Lactate > 5, *n* (%)	12 (17)
Lactate > 10, *n* (%)	3 (4)
Temperature at 4 h	35.8 (34.8, 36.5)
Temperature at 24 h	36.2 (35.4, 36.7)
Plasma exchange, *n* (%)	6 (9)
ECMO duration, h	46 (25, 87)
Post-ECMO EF	2 (1, 3)
Redo cardiac surgery, *n* (%)	2 (3)
Invasive interventions, *n* (%)	5 (7)
Hospital duration, days	41 (26, 57)

**Table 5:** **Incidence of complications**.

Patients with at least 1 complication, *n* (%)	29 (42)
Brain injury, *n* (%)	8 (12)
Seizures, *n* (%)	4 (6)
CNS hemorrhage, *n* (%)	2 (3)
CNS infarct, *n* (%)	2 (3)
Renal dysfunction, *n* (%)	16 (23)
Bloodstream infection, *n* (%)	4 (6)
ECMO support	23 (33)
Oxygenator failure, *n* (%)	4 (6)
Thrombus in ECMO circuit, *n* (%)	9 (13)
Cannula, surgical site, other bleeding, *n* (%)	10 (15)
Respiratory
Pulmonary hemorrhage, *n* (%)	0
Pneumothorax, *n* (%)	1 (1.5)
Cardiac
Arrhythmias on ECMO, *n* (%)	1 (1.5)
CPR on ECMO, *n* (%)	0
Inotropes on ECMO, *n* (%)	7 (10)
Gastrointestinal
Hyperbilirubinemia, *n* (%)	8 (12)

## Description of Outcomes

Discharge and long-term neurodevelopmental outcomes for the entire cohort are presented in Table 6. At hospital discharge, 52 (75%) patients had good – mild disability, and at the latest follow-up this improved to 56 (81%) patients. Six (9%) died between discharge and follow-up. Excluding the patients who died, overall 16 (23%) patients showed improvement in their neurologic status and 3 (4%) showed deterioration. Of the patients who died, one had severe Ebstein’s anomaly and the other five were surgical patients. Three of them had single ventricle anatomy and died after Norwood and prior to the Glenn procedure, one had long standing tetralogy of Fallot and Alagille syndrome in conjunction with end-stage liver disease, and one died after receiving a heart transplant with septicemia.

**Table 6:** **Neurodevelopmental outcomes**.

POPC scale at hospital discharge
Interval time from ECMO, days	35 (18, 47)
Good, *n* (%)	39 (57)
Mild disability, *n* (%)	13 (19)
Mod disability, *n* (%)	12 (17)
Severe disability, *n* (%)	4 (6)
Coma or vegetative, *n* (%)	1 (1)
POPC scale at latest follow-up
Interval time from ECMO, years	1.7 (1, 4)
Good, *n* (%)	42 (61)
Mild disability, *n* (%)	14 (20)
Mod disability, *n* (%)	6 (9)
Severe disability, *n* (%)	1 (1)
Coma, *n* (%)	0
Death, *n* (%)	6 (9)

Comparison of the patients with good – mild disability vs. moderate – severe neurologic disability at hospital discharge and at the latest follow-up is shown in Tables 7 and 8. At hospital discharge more patients with moderate to severe disability had undergone CPR, plasma exchange and had intracranial insult. However, at the latest follow-up, only the presence of cerebral infarction differed between the groups.

**Table 7:** **Comparison of patients with good – mild vs. moderate – severe neurologic disability at hospital discharge**.

	Normal-mild (1–2) POPC *N* = 52 (75%)	Mod-severe (3–6) POPC *N* = 17 (25%)	*p*
Baseline parameters
Cardiac surgery, *n* (%)	35 (67)	9 (53)	0.28
Single ventricle	12 (23)	1 (6)	0.1
Aristotle level 4	21 (40)	3 (18)	0.3
E-CPR, *n*	18 (34)	12 (70)	0.009
CPR duration > 50 min	5 (10)	0	0.01
Pre-ECMO pH < 7.21	7 (13)	4 (23)	0.32
Pre-ECMO lactate > 10	9 (21)	5 (29)	0.5
Chromosomal Abn	5 (10)	2 (12)	0.8
ECMO parameters
ECMO flows > 180	3 (7)	0	0.3
pH < 7.21	2 (4)	0	0.4
Lactate > 10	2 (4)	1 (6)	0.7
Seizures	2 (5)	2 (12)	0.3
Infarct	0	2 (12)	0.009
Hemorrhage	0	2 (12)	0.009
Renal injury	7 (30)	8 (53)	0.2
Plasma exchange	2 (4)	4 (23)	0.01

**Table 8:** **Comparison of patients with good – mild vs. moderate – severe neurologic disability at latest follow-up**.

	Normal-mild (1–2) POPC *N* = 56 (81%)	Mod-severe (3–6) POPC *N* = 13 (19%)	*p*
Baseline parameters
Cardiac surgery, *n* (%)	37 (66)	7 (54)	0.5
Single ventricle	9 (16)	4 (31)	0.2
Aristotle level 4	19 (72)	5 (38)	0.7
E-CPR	24 (43)	6 (46)	0.8
CPR duration > 50 min	5 (9)	0	0.5
Pre-ECMO pH < 7.21	7 (13)	4 (31)	0.2
Pre-ECMO lactate > 10	12 (25)	2 (18)	0.9
Chromosomal Abn	5 (9)	2 (15)	0.6
ECMO parameters
ECMO flows > 180	2 (4)	1 (10)	0.5
pH < 7.21	2 (4)	0	0.8
Lactate > 10	1 (2)	2 (15)	0.1
Seizures	2 (4)	2 (17)	0.2
Infarct	0	2 (15)	0.03
Hemorrhage	2 (4)	0	0.9
Renal injury	11 (35)	4 (57)	0.4
Plasma exchange	4 (7)	2 (15)	0.3

## Discussion

This report represents the neurological outcomes of 69 cardiac ECMO patients at an advanced cardiac ECMO center. Eighty-one percent of the surviving patients had normal to mild neurological impairment at the latest follow-up nearly 2 years after ECMO support, while 19% showed significant neurological impairment (including death). The most significant parameters that appeared to increase risk for poor neurological outcome were presence of CPR, cerebral infarction or hemorrhage, and the use of plasma exchange. Though the latter one likely represents a group of patients who were sicker and thus needing plasma exchange, however we cannot be definitive that the actual procedure itself wasn’t associated more complications and therefore worsening neurologic disability. Our results are similar to the 14% of children who were found to have major neurological impairment (not explained by a chromosomal disorder) at 5 years of age in a multi-center study in Europe and the UK randomized control trial, but less than reported elsewhere (11, 17–19). One important difference with our center compared to the data published elsewhere, is the duration of ECMO averaged <48 h (maximally 87 h), whereas other reports show an average of over 100 h ECMO duration (17, 19). Since ECMO can be associated with so many disastrous complications, we as a group are strong proponents of shortening ECMO duration in the hopes of minimizing complications even at the expense of low to medium doses of inotropic and respiratory support.

Overall it is difficult to determine whether the underlying etiology and cardiac anatomy is more of a significant contributor to the neurological outcomes of patients, or whether the actual course of ECMO itself is more of a significant contributor; particularly with respect to the varying anticoagulation protocols utilized by every center and the common issue of maintaining the target parameters within one’s own protocol. A recent review of the anticoagulation parameters of ECMO patients at our institution over a 5-year period showed that 93% of the recorded high-range ACT (Hemochron ACT+) values were <180 s, which could conceivably contribute to the formation of thromboemboli (9). A recent survey of our ACT data from 2010 until 2013 indicates that 50% of the ACT data are outside the normally accepted range of 180–220 s for the low-range ACT (Hemochron ACT-LR). Ultimately, it may be a combination of the pre-ECMO etiology, which increases risk for poor neurologic outcomes and ECMO complications and then the during ECMO complications (particularly the coagulation status) that determines the composite risk for neurologic injury.

However irrespective of the nature of neurologic injury, this study as well as our previous ECMO survival study (1) show that while superior survival is feasible, neurologic insult can be reduced and that neurologic improvement over the long run is expected. This highlights the importance and investment into early neurodevelopmental intervention for these children.

## Conflict of Interest Statement

The authors declare that the research was conducted in the absence of any commercial or financial relationships that could be construed as a potential conflict of interest.
